# Extracellular Matrix Aggregates from Differentiating Embryoid Bodies as a Scaffold to Support ESC Proliferation and Differentiation

**DOI:** 10.1371/journal.pone.0061856

**Published:** 2013-04-18

**Authors:** Saik-Kia Goh, Phillip Olsen, Ipsita Banerjee

**Affiliations:** 1 Department of Bioengineering, University of Pittsburgh, Pittsburgh, Pennsylvania, United States of America; 2 Department of Chemical and Petroleum Engineering, University of Pittsburgh, Pittsburgh, Pennsylvania, United States of America; 3 McGowan Institute for Regenerative Medicine, University of Pittsburgh, Pittsburgh, Pennsylvania, United States of America; Instituto Butantan, Brazil

## Abstract

Embryonic stem cells (ESCs) have emerged as potential cell sources for tissue engineering and regeneration owing to its virtually unlimited replicative capacity and the potential to differentiate into a variety of cell types. Current differentiation strategies primarily involve various growth factor/inducer/repressor concoctions with less emphasis on the substrate. Developing biomaterials to promote stem cell proliferation and differentiation could aid in the realization of this goal. Extracellular matrix (ECM) components are important physiological regulators, and can provide cues to direct ESC expansion and differentiation. ECM undergoes constant remodeling with surrounding cells to accommodate specific developmental event. In this study, using ESC derived aggregates called embryoid bodies (EB) as a model, we characterized the biological nature of ECM in EB after exposure to different treatments: spontaneously differentiated and retinoic acid treated (denoted as SPT and RA, respectively). Next, we extracted this treatment-specific ECM by detergent decellularization methods (Triton X-100, DOC and SDS are compared). The resulting EB ECM scaffolds were seeded with undifferentiated ESCs using a novel cell seeding strategy, and the behavior of ESCs was studied. Our results showed that the optimized protocol efficiently removes cells while retaining crucial ECM and biochemical components. Decellularized ECM from SPT EB gave rise to a more favorable microenvironment for promoting ESC attachment, proliferation, and early differentiation, compared to native EB and decellularized ECM from RA EB. These findings suggest that various treatment conditions allow the formulation of unique ESC-ECM derived scaffolds to enhance ESC bioactivities, including proliferation and differentiation for tissue regeneration applications.

## Introduction

Embryonic stem cells (ESC) have emerged as an attractive candidate for tissue regeneration owing to its virtually unlimited replicative capacity and potential to differentiate into ∼200 cell types of the human body. One way of *in vitro* differentiation of ESC is to form aggregates called embryoid bodies (EBs), which structurally resemble the pregastrulation-stage embryo [1], [2]. During this stage, temporal expression and spatial distribution of extracellular matrix (ECM) molecules dynamically mediates the differentiation process [3], [4], [5], [6]. For instance, laminin appears as early as the 2-cell stage, entactin/nidogen appears at the 16-cell stage [7], and fibronectin and type IV collagen appears later in the inner cell mass of 3–4 day-old blastocysts [8]. The effects of these ECM proteins in development and morphogenesis have been studied *in vivo* and *in vitro* using gene-knockout animals, over-expression on cells, and surfaces coated with isolated ECM proteins (summarized in review by Rozario et al. [9]). It is hypothesized that these matrices are associated with specific differentiation events, and by recapitulating ECM similar to *in vivo* components will give us more accurate and detailed insights into the role ECM plays in the differentiation of ESC.

Before realization of ESC for regenerative medicine applications, tools must be developed to allow efficient ESC differentiation into specific lineages. While there has been significant progress to understand the role of specific growth factor/inducer/repressor concoctions in inducing differentiation, much effort is being focused to improve the yield and efficiency of lineage specific differentiation. In addition to the role of chemical perturbation, development of biomaterials such as synthetic and natural polymer and hydrogels has also been explored to modulate differentiation of ESC [10], [11], [12], [13], [14]. An avenue which is less explored and only recently gaining momentum, is the effect of native, cell-secreted ECM on cellular differentiation. Since ECM components are critical for cellular differentiation through integrin-mediated activation and downstream signaling events [15] – it can be also be potentially utilized as a tool to modulate ESC differentiation into a specific lineage *in vitro*. Decellularization techniques allow us to extract this complex native ECM, and have been successfully demonstrated in various cells [16], [17], tissues and organs [18]. Decellularized organs such as lung [19], [20], [21] and kidney [22] have been shown to support proliferation and promote site-appropriate differentiation when seeded with mouse embryonic stem cells. In mesenchymal stem cells (MSC), cell secreted ECM *in vitro* can be harnessed via decellularization techniques to yield new cell culture substrates that have been shown to support the regulation of stem cell functions such as proliferation and differentiation [23], [24], [25]. Recently, decellularized matrices from EBs have been developed [26], [27], [28]. It was reported to be a suitable tissue engineering scaffold supportive of fibroblast attachment [27] and further proposed as a naturally-derived ECM to promote wound repair. ECM molecules are synthesized and varied during EB differentiation [5], [6] - these ECM components from differentiating ESC can be considered as a good representation of *in vivo* developmental niche. Hence isolation of these embryonic source ECM molecules could potentially be used as a biomaterial for enhancing ESC differentiation. To this date, the effects of ECM derived from differentiating EB as a scaffold to support ESC functions have not been reported.

In this report we investigated the possibility of utilizing the unique and multifaceted ECM components synthesized by differentiating EBs as a scaffold for stem cell proliferation and differentiation. Toward this end we investigated the differences in synthesized ECM by the EBs exposed to different treatment conditions. Furthermore, the differential effect of such “treatment-specific” ECM from differentiating EBs on stem cells' functions such as proliferation and differentiation were also analyzed.

## Materials and Methods

### Cell culture

The D3 murine ESC line (CRL-1934, ATCC, VA, USA) was maintained on gelatin-coated T75 tissue culture flasks with knock-out Dulbecco's modified Eagle's medium (Life Technologies) supplemented with 15% knockout™ serum replacement, 4 mM Gluta-MAX™ (Life Technologies), 100 U/ml penicillin/streptomycin (Life Technologies), 100 U/ml gentamicin (Life Technologies),1000 U/ml leukemia inhibitory factor (LIF; EMD Milipore) and 0.1 mM 2-mercaptoethanol (Life Technologies). The Nagy ES cell line R1 with EGFP (B5) (ES-R1-EGFP B5/EGFP cells; purchased from MMRRC repository, University of Missouri) [29] was cultured in 15% replacement serum, 2 mM GlutaMAX™ (Life Technologies), 50 U/ml penicillin (Life Technologies), 0.1 mM MEM Non-essential Amino-acids (NEAA; Life Technologies), 1 mM Sodium Pyruvate (Life Technologies), 1000 U/ml leukemia inhibitory factor (LIF; EMD Milipore) and 0.1 mM 2-mercaptoethanol (Life Technologies) on gelatin-coated T75 tissue culture flasks. Both cell types were cultured at 37°C and in a 95% air/5% CO_2_ atmosphere.

### Embryoid Bodies formation and differentiation

Undifferentiated D3 murine ES cells were trypsinized to form a single-cell suspension of 1×10^6^ cells/ml density. Embryoid bodies (EB) formation was initiated by transferring cells into 2 ml of culture medium (Iscove's Modified Dulbecco's Medium (Life Technologies) supplemented with 20% fetal bovine serum (FBS; Atlanta Biologicals), 1% Penicillin-Streptomycin (Life Technologies), and 10 µg/ml gentamicin (Life Technologies)) in 35 mm×10 mm non-tissue culture-treated petri dishes. Dishes were placed on orbital shaker (Rotamax 120, Heidolph) in an incubator with 37°C and 95% air/5% CO_2_ atmosphere. The orbital shaker was maintained at 40 rpm as described by another study [30]. Starting from day 2, culture medium was changed daily. For the retinoic acid (RA) treated group, 10^−7^ M of RA was supplemented to the culture medium at this time. For the spontaneously (SPT) differentiated EB group, only the culture medium was added.

### Fabrication of EB ECM scaffolds via decellularization

**Figure 1:**
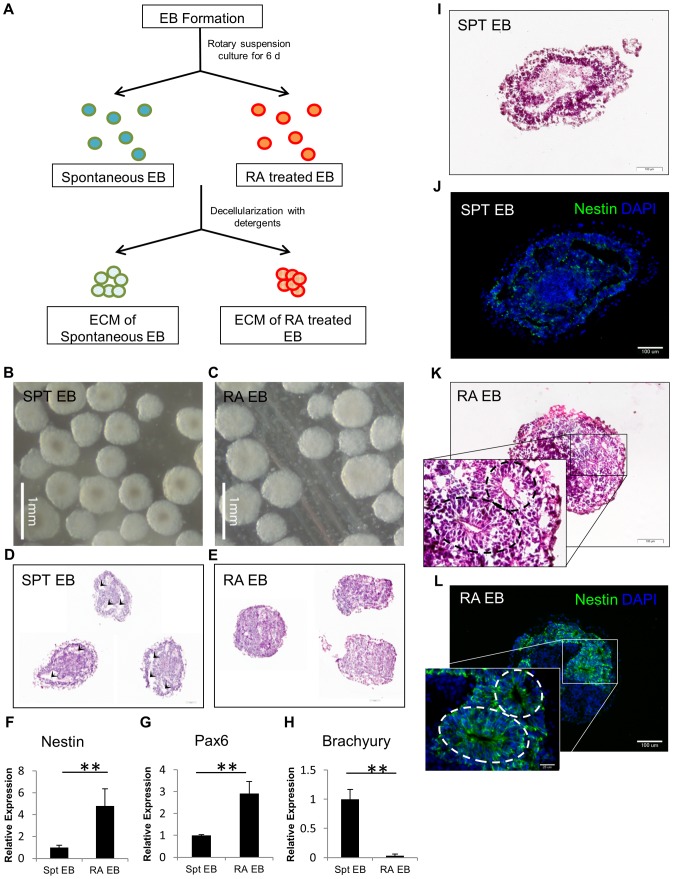
EB preparation, morphology and gene analysis. (A) Preparation scheme for EB derived ECM scaffold. (B–C) Generation of EB via rotary suspension culture resulted in homogenous-sized EBs. Histology showing both groups of native EB – (D) SPT EB and (E) RA treated EB. Arrow heads indicate numerous cavities within the SPT EBs more than RA treated EB. (F–H) Quantification of gene expression via qRT-PCR shows that RA induces neural differentiation of EBs. (F) Nestin, (G) Pax6, and (H) Brachyury. Relative expression is normalized to SPT EB. H&E staining of EB sections shows presence of neural rosettes (dotted lines) in the (K) RA treated EBs confirming neuroepithelial tissue formation in contrast to (I) SPT EB. Immunohistochemical analysis of consecutive sections demonstrated positive for anti-Nestin staining in (J) RA treated EB but minimally expressed in (L) SPT EB. All values are mean ± SD, p<0.01(**), n = 3, represents pooled of EBs from 3 experimental repeats.

After 6 days of rotary suspension culture, EBs were collected and divided into individual samples in 1.5 ml microcentrifuge tubes using two plates of EBs to form one respective EB scaffold. Two groups of EB ECM scaffolds were produced as outlined in Figure 1A. EBs were decellularized by chemical detergent washing. Three types of detergent were evaluated – one non-ionic detergent, 1) 1% TritonX-100 and two ionic detergents, 2) 0.1% Sodium Dodecyl Sulfate (SDS), 3) 0.1% Sodium Deoxycholate (DOC) diluted in deionized water. 800 µl of detergents were added to each sample and placed on 3-D rotator (Lab Line, Thermo Scientific) with continuous rotation for 30 min at room temperature. Individual samples were spun down at 18,000 g for 2 min and washed twice with PBS according to the same EB decellularization protocol as Nair et al. [28]. The resultant decellularized EB scaffolds were suspended in PBS supplemented with 5% Pen/Strep and stored at 4°C before further applications.

### Immunostaining and Histology

We fixed and sectioned native and decellularized EBs following standard protocol. Briefly, EBs were fixed with 4% formaldehyde (Thermofisher) overnight and cryoprotected with 30% sucrose for 48 hours before embedded in OCT for cryo-sectioning. Samples were cut into 7 µm thick sections. H&E staining was first done to determine gross morphology of EBs pre and post detergent treatment. For immuno-staining, slides were first rehydrated in 2 washes of PBS (Fisher). Next, tissue samples were permeabilized with 0.1% Triton-X in PBS for 10 min. A blocking step with 10% donkey serum in PBS for 45 min was done before antibody incubation. For primary antibodies, we used the following antibodies: rabbit anti-laminin, rabbit anti-collagen I, rabbit anti-collagen IV, rabbit anti-fibronectin, and rabbit anti-Brachyury (Abcam, 1∶200). Incubation time for primary antibodies was overnight in 4°C. Before secondary antibodies incubation, we washed slides three times (5–10 min) each with 1× PBS. The secondary antibody used was: donkey anti-rabbit Alexafluor 488 (1∶500, Invitrogen). Incubation time for secondary antibody was 45 min in room temperature. The slides were washed again three times with 1× PBS (5–10 min) each before being covered with hardening mounting medium containing DAPI (Vectashield, Vector laboratory). Quantification of mean fluorescence intensity (MFI) of ECM staining per EB was accomplished by converting all images to grayscale followed by analysis with Metamorph Image software. Similarly for Brachyury quantification, mean fluorescence intensity (MFI) per nucleus per field was done.

### Scanning Electron Microscopy

We fixed the native EBs and decellularized EB scaffolds samples in 2.5% glutaraldehyde in 0.1 M PBS (pH 7.4) for 60 minutes. The samples were washed thoroughly in 3 changes 0.1 M PBS for 15 minutes each. Next, the samples were fixed in 1% OsO4 in 0.1 M PBS for 60 minutes. This was followed by another 3 changes of PBS washing steps for 15 minutes each. The samples were then dehydrated in gradient series of alcohol for 15 min each. Additionally, samples were critical point dried and coated with Au/Pd using a Cressington Coater 108A sputter coater. Electron microscope images were taken using a Jeol JSM-6335F field emission SEM.

### DNA, sGAG, Protein and Collagen Quantification

The decellularized EB scaffolds (n = 3, individual EB scaffold) were digested with papain solution at 60°C for 6 h. The pooled native EBs before decellularization (n = 3) were digested in papain solution as controls. Papain (Sigma Aldrich) was dissolved at 400 mg/ml in 0.1 M phosphate buffer (pH 6.0), with 5 mM cysteine hydrochloride (Sigma Aldrich), and 5 mM EDTA (Sigma Aldrich). The lysates were used for detection of the DNA and sulfated glycosaminoglycan (sGAG) content. A DNA quantification kit - Quant-iT™ PicoGreen® dsDNA Assay kit (Invitrogen) was used to measure DNA content according to manufacturer's instruction. The fluorescence reading (excitation: 485 nm and emission: 528 nm) was taken on a plate reader (Synergy 2, Biotek), and the absolute amount of DNA (ng/mL) was quantified against a lambda DNA standard curve (0 ng/mL–1000 ng/mL).

The sGAG amounts were measured by a Blyscan sGAG assay kit (Biocolor) according to the manufacturer's manual. Briefly, the sample lysate was mixed with Blyscan dye to bind the GAG. The GAG-dye complex was then collected by centrifugation. After the supernatant was removed and the tube drained, the dissociation reagent was added. The absorbance against the background control was obtained at a wavelength of 656 nm on a microplate spectrophotometer and the GAG amount (n = 3, individual EB scaffold) was calculated based on a standard curve obtained with the standard GAG supplied with the kit.

For protein and collagen measurement, the native and decellularized EBs were dissolved in 0.5 M acetic acid containing 0.1 mg/ml pepsin for 48 h at 48°C. For protein quantification, BCA protein assay (Thermofisher) was done according to manufacturer's instruction. Briefly, the dissolved samples were incubated in the BCA solution for 30 min at 37°C, and the absorbance readings were taken at 562 nm on a plate reader. The concentrations were determined by comparing experimental values to standard curve readings obtained using BSA titration standard curve.

The collagen content was determined using Sircol Collagen assay (Biocolor) according to manufacturer's instructions. Briefly, the sample lysate was mixed with Sircol dye reagent, and the collagen-dye complexes formed and precipitated out from the soluble unbound dye. The pellet was washed once with Acid-Salt wash reagent and suspended in alkali reagent. The absorbance was read at 555 nm and the amount of collagen was calculated based on a standard curve obtained using the standard control of bovine type 1 collagen supplied in the kit.

### Cell Seeding and *In-vitro* Culture

Undifferentiated R1 murine ES cells with EGFP (B5) were trypsinized to form a single-cell suspension. Three different methods were employed to analyze ESC attachment on the decellularized EB scaffolds. (1) *Static method*: R1 ESC were seeded on decellularized EB scaffolds in 35 mm×10 mm non-tissue culture-treated petri dishes, and then placed in incubator for 6 hours at 37°C; (2) *Dynamic orbital shaker method*: The procedure was similar to the static method, except that the cell-seeded EB scaffolds containing plates were placed on a shaking platform at 40 rpm for 6 hours during the incubation period; (3) *Hanging drop seeding method*: The decellularized EB scaffolds were seeded by suspending 50 µL of cell suspension together with the ECM scaffolds on the lid of 100 mm×15 mm dishes for 6 hours during the incubation period. All three methods were seeded with the same cell density of 0.1×10^6^ cells/ml in differentiation medium consists of ESC growth medium without LIF. After the seeding steps, the cell-seeded EB scaffolds were cultured in 35 mm×10 mm non-tissue culture-treated dishes under static condition for 6 days with medium changed every 1–2 days.

### Two-photon Microscopy

Two-photon microscopy was done with an upright Olympus FV1000 MPE multiphoton microscope (Olympus, Central Valley, PA, USA) and a Mai Tai DeepSee femtosecond-pulsed laser (Spectra-Physics, Santa Clara, CA, USA) tuned at 800 nm. The cell-seeded EB scaffold was line-scanned and fluorescence emission was captured by three nondescanned external photomultiplier tube (PMT) detectors coupled to the following longpass dichroic mirrors and bandpass emission filters: 505 nm mirror and 460–500 nm filter (blue channel), 570 nm mirror and 520–560 nm filter (green channel) and 575–630 nm filter (red channel). The cell-seeded EB scaffold was placed on an imaging dish having a #1.5 coverslip and immersed in PBS. Fixed *xy* planes spanning 505×375 µm at a resolution of 0.994 µm/pixel and depth of 1–200 µm from the surface of scaffold were imaged using a high numerical aperture (NA = 1.05), water-immersion 25× objective.

### Viability and Live/dead Assay

Cell viability was quantified by assessing the cell metabolic activity using Alamar Blue assay (Invitrogen) according to manufacturer's instruction. Briefly, 1 to 10 dilution of Alamar Blue reagent was diluted to the culture medium and added to the cell-laden scaffold for up to 4 hours. Samples of culture medium were then collected and fluorescence readings (excitation: 485 nm and emission: 528 nm) were taken on a plate reader (Synergy 2, Biotek), and the value was quantified against the control EB scaffold without cells added. For Live/dead assay (Invitrogen), murine ESC D3 line were used for reseeding to assess the cytocompatibility of the EB scaffold. Briefly, calcein AM (1 µM) and EthD-1 (2 µM) were added together with culture medium to cell-laden scaffolds and incubated for 15 minutes under light protection in room temperature. Samples were washed twice with PBS and continued with epifluorescence microscopy on the whole mounts cell-seeded scaffold to distinguish between live cells (green) and dead cells (red).

### Quantitative RT-PCR

RNA was extracted using NucleoSpin kit according to the manufacturer's protocol. The sample absorbance at 280 nm and 260 nm was measured using a BioRad Smart Spec spectrophotometer to obtain RNA concentration and quality. Reverse transcription was performed using ImProm II Promega reverse transcription kit following the manufacturer's recommendation. qRT-PCR analysis was performed for early germ layer markers, Brachyury (primitive streak and mesoderm), FGF8 (epiblast and mesoderm), FGF5 (epiblast), Nestin (neuroectoderm), PAX6 (neuroectoderm), AFP (visceral endoderm) and pluripotency maker, Nanog.

The cycle number at the threshold level of log-based fluorescence is defined as Ct number, which is the observed value in most real-time PCR experiments, and therefore the primary statistical metric of interest. ΔCt is equal to the difference in threshold cycle for target and reference or control (ΔCt = Ct_target_−Ct_reference_). ΔΔCt is equal to the difference between ΔCt_sample_ and ΔCt_control_ (ΔΔCt = ΔCt_sample_−ΔCt_control_). The fold change of a target gene is defined by, fold change = 2^−ΔΔCt^. qRT-PCR analysis was repeated in triplicate.

### Statistics

Quantification data were expressed as mean ± SD. Significant differences among groups were determined by two-tailed Student's t-test for two-group comparisons or ANOVA followed by post-hoc analysis for multiple group comparisons. Probability values at P<0.05 (*) and P<0.01 (**) indicated statistical significance.

## Results

### Physiochemical differences in spontaneously differentiated EB and RA treated EB

Embryoid Bodies (EB) were formed from murine ESC D3 line by rotary suspension cultures, initiated at a density of 1×10^6^ cells/ml. Uniform and spherical shaped EBs were noticed after 2 days of suspension culture as it has been shown in previously [30]. From day 2 onwards, the first group of EB was treated with 10^−7^ M retinoic acid (RA) to induce neuronal differentiation and the second group was spontaneously differentiated (SPT) without RA treatment. Nestin was used as a marker to assess neuronal differentiation and the optimal RA concentration to give rise to highest Nestin expression was determined to be 10^−7^ M upon testing a series of RA dilution (10^−5^ M to 10^−7^ M) (Figure S1). After 6 days, both groups of EBs were harvested for analysis. From morphometric analysis, both groups of EBs increased in size (∼450 µm) and remained largely homogenous in shape (Figure 1B, C). H&E staining demonstrated the formation of cystic cavities in the SPT EBs whereas there is little evidence of cavitation in the RA EBs (Figure 1D, E). To elucidate the effect of RA towards the germ layer commitment and differentiation of EB, we first analyzed the gene expression profiles of the EBs using qRT-PCR. RA treated group demonstrated 4.8-fold and 2.9-fold upregulation in neural progenitor maker, Nestin and neuroectoderm marker, Pax6 respectively and strong repression of mesodermal genes such as Brachyury compared to SPT EB (Figure 1F–H). In addition, neuroectodermal precursors in neural rosettes were observed in H&E staining of RA treated EB (Figure 1K). The differentiation was also confirmed by presence of neuroepithelial tissues by Nestin positive staining from immunohistochemical evaluation (Figure 1L) of the RA treated EB. In contrast, very low Nestin expression was detected in the SPT EB (Figure 1J). These results suggest that RA treatment of EB is promoting neuroectoderm differentiation of ESC.

**Figure 2:**
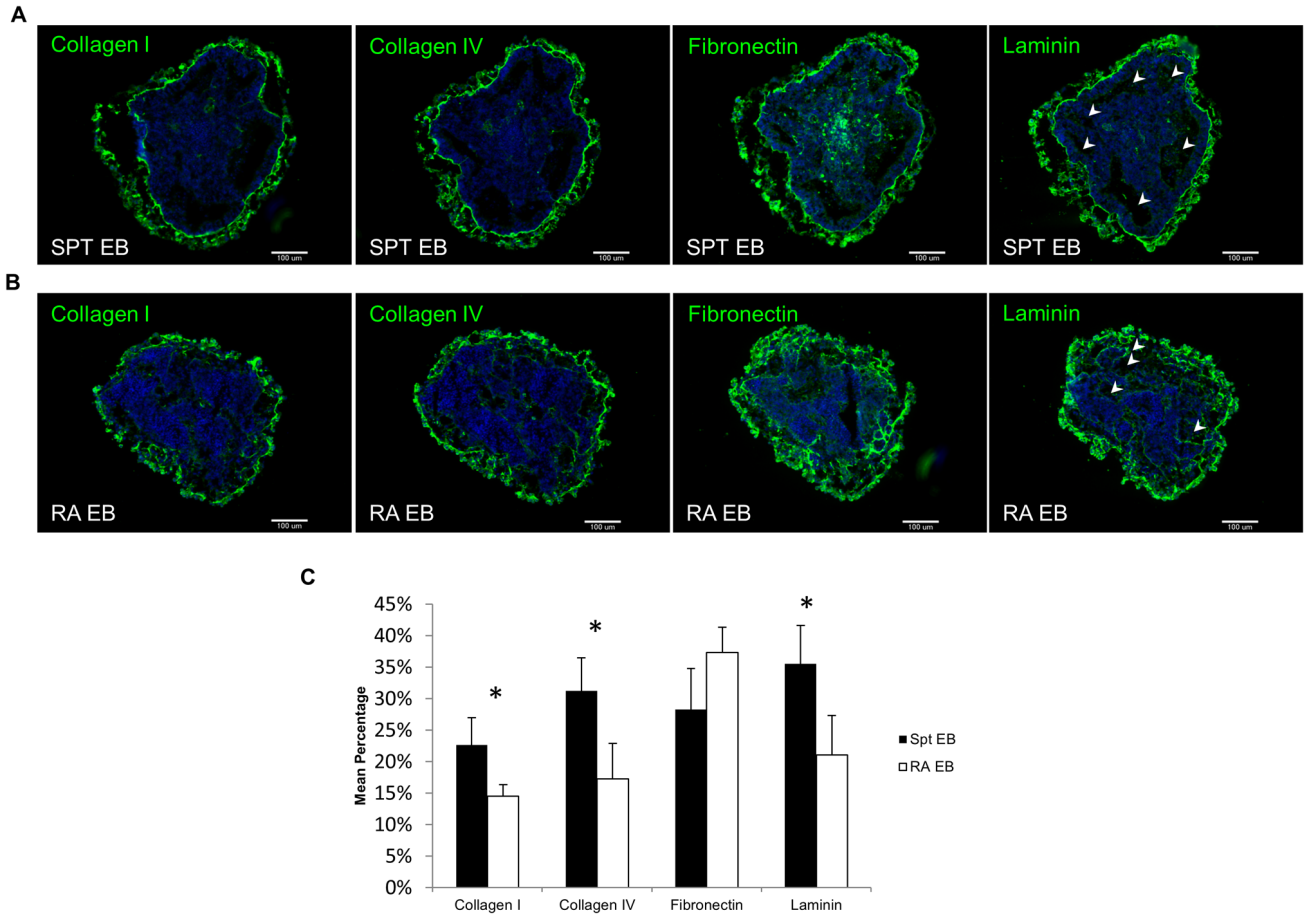
ECM characterization of native EBs resulting from different treatment - SPT vs. RA. Immunofluorescence images of both groups of EBs – (A) SPT and (B) RA composing ECM molecules. Arrow heads indicate more cavities were found within the SPT EBs than the RA EBs. (C) Metamorph image analysis showing the quantification of the mean fluorescence intensity (MFI) of ECM markers stained in both group of EBs. Bar = 100 µm. All values are mean ± SD, p<0.05 (*), n = 6, represents pooled of EBs from 6 experimental repeats.

To evaluate whether SPT EB have a different ECM composition than RA induced EB, we characterized the ECM components of the two groups of EB by immunohistochemical (IHC) analysis. Four different ECM proteins of interests were evaluated: Collagen I, Collagen IV, Fibronectin and Laminin. All four classes of ECM proteins were detected in both groups of EBs (Figure 2A, B). Quantitative image analysis of IHC staining using Metamorph software demonstrated that the mean percentage of Collagen I, IV and Laminin expressions were higher in SPT EBs than the RA EBs (P<0.05) (Figure 2C). While the mean percentage of Fibronectin expression showed insignificant difference between the two groups of EBs (Figure 2C), distinct difference in spatial distribution of fibronectin was observed between the two groups. Apart from the outer periphery, strong fibronectin expression was also detected in the inner core of SPT EB. In contrast, for the RA treated EB group fibronectin was detected predominantly around the peripheral region (Figure 2A, B). These results depict the physiochemical differences in terms of ECM proteins, morphometric and gene expression profiles between RA treated EB compared to SPT EB.

### Cell removal via detergent decellularization

**Figure 3:**
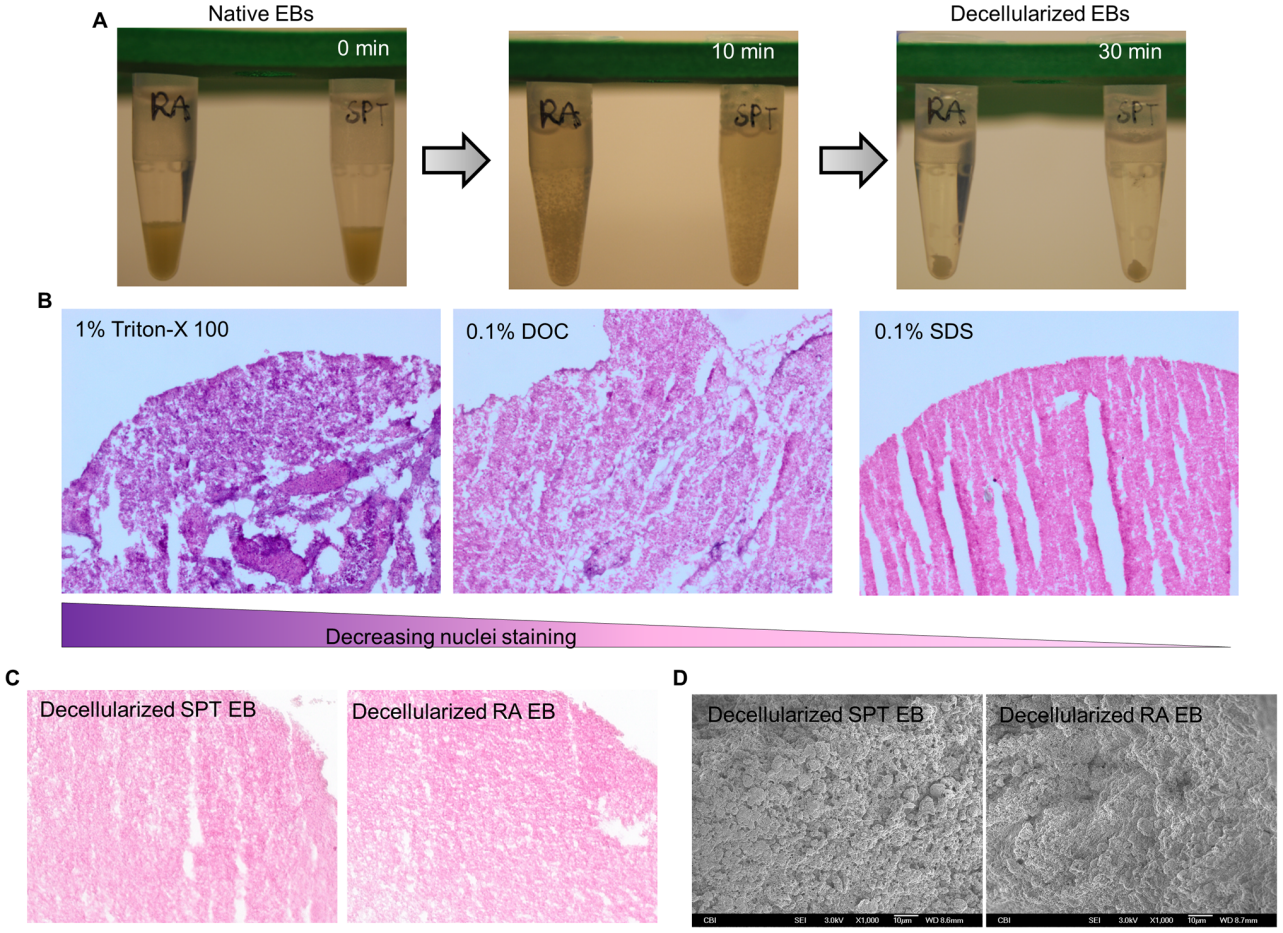
Decellularization of SPT and RA treated embryoid bodies. (A) Panel images depict the decellularization process of EB. (B) Histological analysis of decellularized EB by H&E staining to show cell removal efficiency with three different detergents. (C) H&E staining of both groups of decellularized EB scaffolds showing absence of intact nuclei. (D) SEM images after decellularization process shows dense particulate material without distinct individual cell in both groups of EBs.

Cell removal technique via detergent is an effective strategy to isolate and preserve ECM components in the native organs and tissues, and have also been demonstrated in EBs [26], [28]. We evaluated the effectiveness of three different detergents (1% Triton X-100, 0.1% DOC, and 0.1% SDS) on both groups of EBs. EBs were first distributed equally into microcentrifuge tubes before addition of detergents. After the decellularization process, the resulting decellularized EB lost their individual EB structure but compacted to form an agglomerated mass of ECM (Figure 3A). From gross H&E examination, 1% Triton X-100 treated EBs gave highest intensity of hematoxylin staining but 0.1% DOC resulted in some punctate hematoxylin staining. The third detergent examined, 0.1% SDS, appeared to be the most effective detergent in complete cell removal as shown by lack of hematoxylin nuclei staining (Figure 3B). Thus, in all the results described hereafter 0.1% SDS was used as the cell removal detergent due to the high efficiency of cellular material removal.

### Preservation and characterization of ultrastructure, ECM and biochemical components after decellularization

The two groups of EBs were further compared and characterized for preservation of ultrastructure and ECM composition after decellularization by 0.1% SDS treatment. First of all, analysis of histological sections confirmed removal of intact nuclei from both groups of EBs (Figure 3C). The ultrastructure of the decellularized EB scaffold of both groups was analyzed by scanning electron microscopy (SEM). Both groups showed comparable ultrastructural morphologies with preservation of structures such as grooves, ridges, and fibrillar meshwork of ECM (Figure 3D). However, no distinct individual cells were detected with SEM. DNA quantification via Picogreen analysis further confirmed DNA removal (<1% DNA content remained) in both groups (Table 1). These results suggest that the decellularization technique successfully removed cellular content.

**Table 1:** DNA content via Picogreen assay.

	*DNA Content (µg/EB scaffold)*	
	*Native (Pre)*	*Decellularized (Post)*
**SPT EB**	27.30±1.30*	0.09±0.04
**RA EB**	25.21±1.40*	0.07±0.04

Data represent means ± S.D. (n = 3), represents pooled of EBs from 3 experimental repeats.

* Significant difference compared with decellularized EB scaffold, P<0.01.

**Figure 4:**
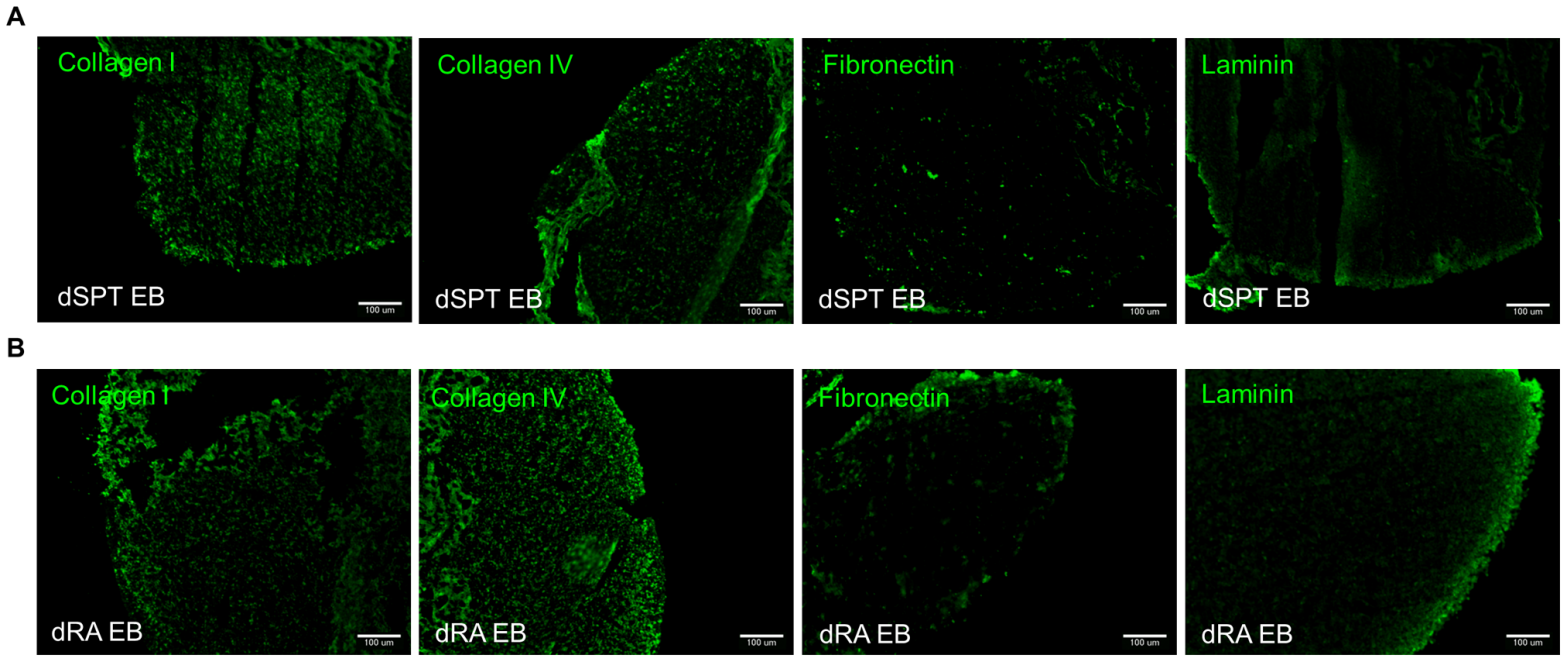
Immunofluorescence images of ECM biomolecules preserved after decellularization treatment in both groups of EBs. (A) dSPT EB and (B) dRA EB. Both groups of decellularized EB preserved Collagen I, Collagen IV, Fibronectin, and Laminin after decellularization treatment.

Preservation of ECM components was evaluated by performing IHC on the same four ECM markers examined on the native EBs (Col I, Col IV, Fibronectin and Laminin). IHC confirmed the retention of all four components of ECM proteins in the decellularized EBs (Figure 4A, B). Absence of DAPI staining confirmed cellular content removal (Figure 4A, B). These results confirm the effectiveness of 0.1% SDS in removing the cellular components with preservation of ECM proteins. However it is worth noting that the process of decellularization is unable to preserve the differences in ECM composition and spatial distribution between the two groups that were detectable in their native form.

To quantify the retention of total protein mass after decellularization in the two groups of EBs, BCA protein assay was used. Total protein content in decellularized spontaneous (dSPT) and decellularized RA treated (dRA) EB was 30% and 21% of the native protein content, respectively (Table 2). A colorimetric Blyscan assay was used to quantitatively analyze of sulfated glycosaminoglycans (sGAG) content. The sGAG content was retained in both decellularized EB scaffolds (Table 3), although was significantly reduced upon decellularization (P<0.01). dSPT retained a higher sGAG content than the dRA (P<0.01). Sircol Collagen assay content in dSPT and dRA EB demonstrated the collagen level decreased to 47% and 53% respectively after decellularization. However, the resulting dSPT EB scaffold had higher collagen content than the dRA EB scaffold (P<0.05, Table 4). Collectively, these results suggest that decellularization process successfully retained the bioactive ECM components.

**Table 2:** Total protein content analysis via BCA assay.

	*Total Protein Content (µg/EB scaffold)*	
	*Native (Pre)*	*Decellularized (Post)*
**SPT EB**	1538.63±209.97*	462.79±30.72
**RA EB**	1477.28±281.58*	304.93±23.38

Data represent means ± S.D. (n = 3) represents pooled of EBs from 3 experimental repeats.

* Significant difference compared with decellularized EB scaffold, P<0.01.

**Table 3:** sGAG content via Blyscan assay.

	*sGAG Content (µg/EB scaffold)*	
	*Native (Pre)*	*Decellularized (Post)*
**SPT EB**	91.33±16.04*	1.00±0.30
**RA EB**	83.45±7.55*	0.76±0.12

Data represent means ± S.D. (n = 3) represents pooled of EBs from 3 experimental repeats.

* Significant difference compared with decellularized EB scaffold, P<0.01.

**Table 4:** Collagen content via Sircol Collagen assay.

	*Collagen Content (µg/EB scaffold)*	
	*Native (Pre)*	*Decellularized (Post)*
**SPT EB**	461.17±89.04*	216.67±43.68
**RA EB**	265.86±58.55*	140.91±33.96

Data represent means ± S.D. (n = 3) represents pooled of EBs from 3 experimental repeats.

* Significant difference compared with decellularized EB scaffold, P<0.01.

### Seeding of ESC on Decellularized EB Scaffolds

**Figure 5:**
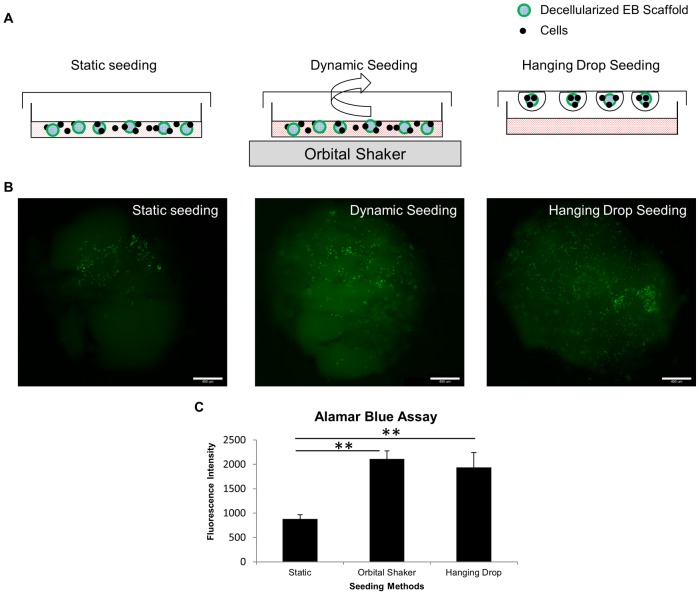
Analysis of seeding efficiency of EB scaffolds by three different seeding methods. (A) Schematic illustration to demonstrate three different methods of cell seeding. (B) Whole mount fluorescence images of seeded EB scaffolds demonstrate three different seeding strategies on decellularized EB scaffolds examined at 6 hours after initial seeding. (C) Alamar Blue assay depicts higher seeding efficiency in both the orbital shaker and hanging drop seeding method than the static method. Bar = 450 µm. All values are mean ± SD, p<0.01 (**), n = 3, represents individual seeded EBs in 3 experimental repeats.

To evaluate the potential of ECM derived from decellularized EBs to be used as a biologic scaffold, we seeded both groups of decellularized EB with eGFP-tagged mouse ESC (ESR1 eGFP). Effective cell seeding throughout the scaffold often determines the success of tissue engineering applications, hence we evaluated 3 different cell seeding methods: 1) static, 2) dynamic (orbital shaker), and 3) hanging drop seeding (shown in schematic Figure 5A) with same cell seeding density (0.1×10^6^ cells/ml). The initial attachment efficiency was analyzed by epifluorescence microscopy after 6 hours of seeding (Figure 5B). Feeble auto-fluorescence signal in the decellularized ECM under FITC channel (green) was used to distinguish the decellularized ECM from the bright exogenously seeded EGFP-labeled cells. All three seeding strategies resulted in cellular attachment, although with varying efficiency. Dynamic and hanging drop seeding methods resulted in highest efficiency and static seeding method the lowest. These observations were further confirmed by quantitative Alamar Blue assay at 6 hours after seeding. Higher metabolic activity observed in EB scaffolds seeded by orbital and hanging drop seeding methods (P<0.01) suggests higher cell attachment efficiency than the static seeding method. No significant difference was found between the dynamic and hanging drop seeding method (Figure 5C). Hanging drop method of cell seeding was thus utilized for the all the experiments hereafter because of better control over cell number/ECM scaffold during seeding process.

### Proliferation, survival and *in-vitro* culture of cell-seeded decellularized EB scaffolds

**Figure 6:**
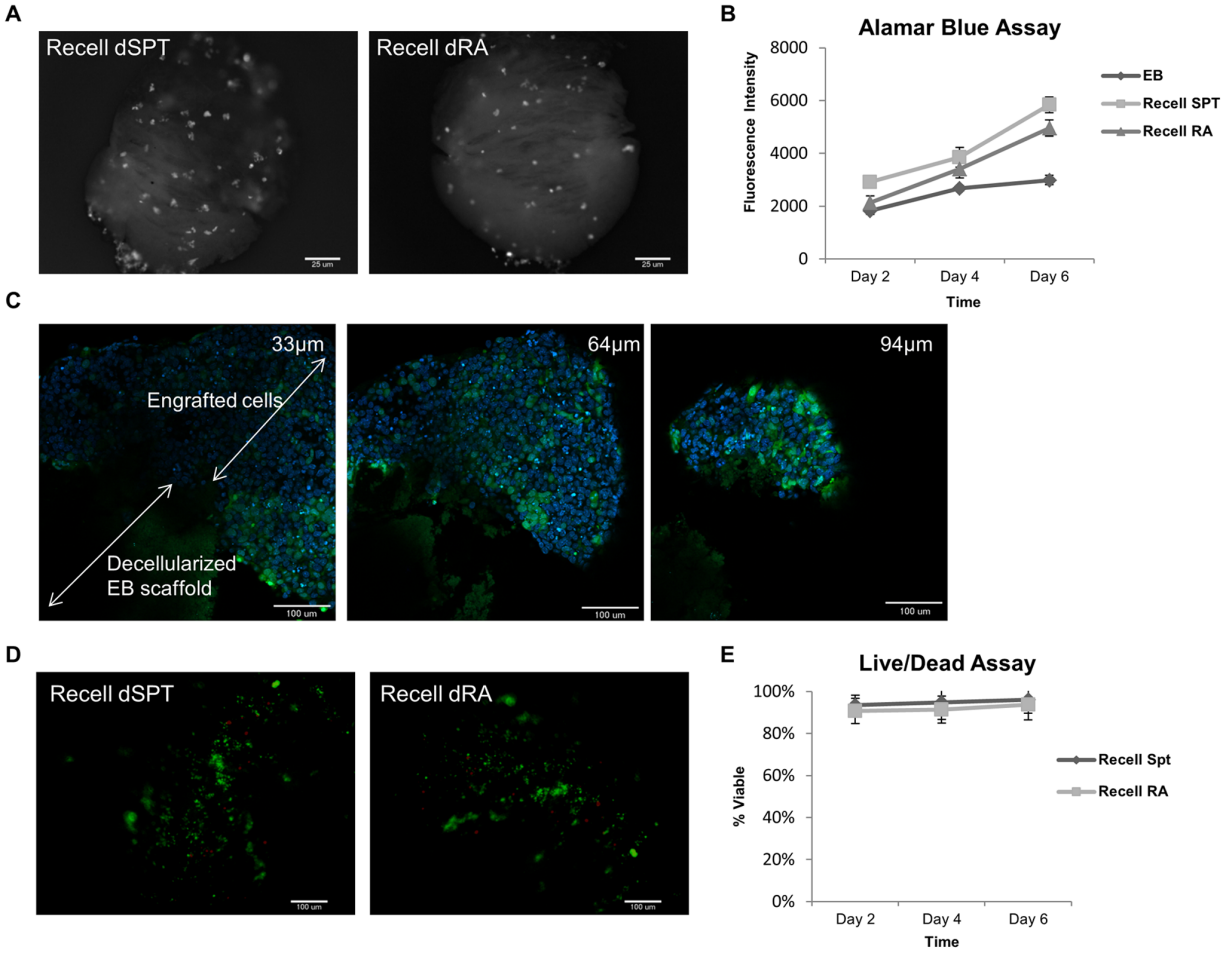
Examination of viability, engraftment location and proliferation kinetics of the seeded EB constructs. (A) Whole mount fluorescence images of seeded EB scaffolds at day 2 depict more ESR1 cells attach and grow on dSPT than dRA EB scaffolds. (B) Alamar blue assay depicts higher proliferation of ESR1 seeded on both decellularized EB scaffolds compared to native ESR1 EB over the course of 6 days. (C) Representative live/dead staining images show the survival of the engrafted ESR1 cells on both dSPT and dRA EB scaffolds after 2 days of culture. (D) Image analysis of live/dead assay to depict the mean percentage of live cells. (E) XY single-plane-two-photon imaging of cell-seeded constructs was done at depths of 33, 64 and 94 µm – this demonstrates that engrafted cells attached on the surface of the scaffolds with minimal infiltration into the ECM scaffold. All values are mean ± SD, n = 3, represents individual seeded EBs in 3 experimental repeats.

Following seeding of ESR1 on both groups of decellularized EBs, the cell-laden scaffolds were cultured for 6 days in suspension on non-tissue culture treated plates under static conditions. At day 2, more cells were found on dSPT EB scaffold than dRA EB scaffold, as observed by epifluorescence microscopy (Figure 6A). Proliferation of the seeded ESR1 on both groups of decellularized EB scaffolds were monitored via alamar blue colorimetric assay and compared to proliferation kinetics of native ESR1 EB. At day 2, alamar blue assay was consistent with epifluorescence miscroscopy observation suggesting more cells attached and growing on dSPT EB scaffold (Figure 6B). Over the course of 6 days culture time, both groups of seeded decellularized EB demonstrated increased proliferation with higher proliferation observed on cells seeded on the dSPT EB scaffold. In contrast, proliferation on native ESR1 EBs was leveling off at day 4 onwards (Figure 6B).

In order to better evaluate the cellular location with respect to the engraftment matrix, we visualized the cell-ECM construct with multi-photon microscopy, 6 days after culture. We observed that the engrafted ESR1 proliferates outward from the decellularized EB scaffolds. Minimal cellular infiltration was observed into the decellularized EB matrices (Figure 6C).

To demonstrate the cytocompatibility of the EB scaffold, cell viability was determined by live/dead assay. After 2 days of culture, only a few dead cells were found on both the seeded constructs (Figure 6D). The percentage of viable cells attached on both decellularized EB scaffolds was greater than 90% for the 6 days of culture period (Figure 6E). These results suggest that the ECM proteins from decellularized EBs (exogenous ECM) positively impacted the ESR1 growth, with the ECM from SPT EB providing a more favorable ECM microenvironment in terms of cell attachment and proliferation.

### ECM from decellularized EB scaffolds stimulates early differentiation of ESC

Cell-ECM interaction is critically important in patterning and morphogenesis of the gastrulating mouse embryo [31], [32]. Since ECM scaffolds from both decellularized groups of EB were supportive of ESC attachment and proliferation, we next investigated the effects of the ECM towards differentiation of ESC. After 6 days of culturing ESC on both the decellularized EB scaffolds, cells were harvested and analyzed for gene expression by qRT-PCR. Native differentiating EB from ESR1 were used as control group for comparison. Undifferentiated ESR1 were also compared (Figure S2). We specifically concentrated on early differentiation markers - Brachyury (primitive streak and mesoderm), FGF8 (epiblast and mesoderm), FGF5 (epiblast), AFP (visceral endoderm), Nestin (neuroectoderm) and also pluripotency maker, Nanog.

**Figure 7:**
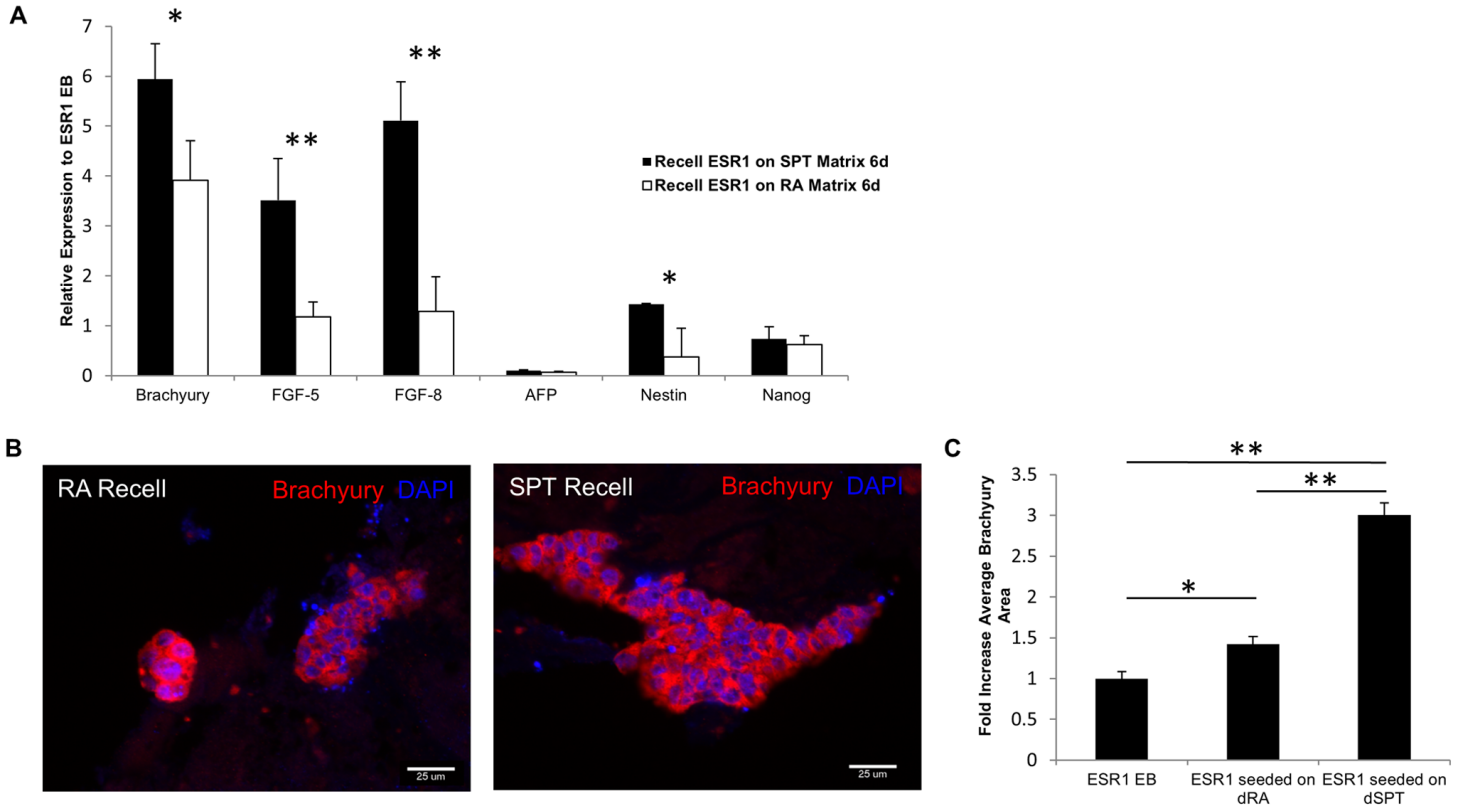
Gene and protein expression of the seeded EB constructs after 6 days of culture. (A) qRT-PCR result at day 6 to depict gene expressions related to early gastrulation. The expression level is normalized to native ESR1 EB at day 6. Higher early differentiation markers –Brachyury, FGF5 and FGF8 were found on ESR1 seeded on dSPT EB scaffold. VE gene – AFP and pluripotency gene – Nanog are downregulated on both seeded constructs compared to native EB. Neuroectoderm marker – Nestin was upregulated on seeded dSPT EB scaffold but downregulated in seeded dRA EB scaffold. (B) Brachyury protein expression was investigated and confirmed by IHC – representative images showing higher cell numbers found positive for Brachyury in ESR1 seeded on dSPT EB scaffold compared to dRA EB scaffold. (C) Cell number area positive for Brachyury were measured and compared among ESR1 seeded on dSPT, dRA EB scaffolds and native ESR1 EB and average Brachyury immunoreactive area was significantly greater on ESR1 seeded on dSPT scaffold. All values are mean ± SD; p<0.05 (*), P<0.01 (**), n = 3, represents individual seeded EBs in 3 experimental repeats.

The primitive streak marker Brachyury was expressed an average of 5.9-fold and 3.9-fold higher on dSPT and dRA EB scaffolds respectively, compared to the native, spontaneously differentiating EB - control (Figure 7A, P<0.05). On dSPT cell seeded constructs, FGF5 which is an epiblast gene showed 3.5-fold higher average upregulation than that of native EB, however there wasn't any significant difference found in dRA cell seeded construct compared to native EB (Figure 7A, P<0.01). FGF8 is expressed in pre-gastrulation epiblast and then in the primitive streak on the mesoderm front [33] - it was found to express an average of 5.1-fold higher on dSPT cell seeded constructs (Figure 7A (P<0.01). Neuroectoderm – Nestin expression was found to be an average 1.4-fold higher (P<0.05) on dSPT cell seeded construct but downregulated on dRA cell seeded construct.

Our qRT-PCR analysis of the 3 groups of cells revealed distinct differences between both (i) the native EB and the cell-ECM constructs and (ii) in between the two groups of decellularized ECM. The strongest differences were observed between the cell-dSPT construct and the native EB as well as the cell-dSPT and cell-dRA construct. Quite interestingly, differences between native EB and cell-dRA construct were insignificant, except for the primitive streak marker Brachyury, which was strongly upregulated in both the cell-ECM constructs as compared to native EB.

Comparison between the two groups of cell-ECM construct revealed that all of the tested markers were significantly stronger in the cell-dSPT construct as compared to either cell-dRA construct or native EB. The only exception was VE marker; AFP which was downregulated in both cell-ECM constructs (Figure 7A). No significant difference of FGF8 and Nestin expression was detected between dRA cell seeded construct and native EB. Pluripotency marker Nanog was found to decrease in both the dSPT and dRA cell seeded constructs (Figure 7A).

To further characterize the differentiated cells on both cell-ECM constructs, we performed immunostaining. The cells seeded onto the dSPT and dRA ECM scaffolds were stained positive for Brachyury. We observed more cells found positive for Brachyury in the dSPT cell-seeded group. Number of cell area stained by IHC demonstrated an average of 3.0-fold and 1.4-fold higher of brachyury expression on dSPT and dRA EB scaffolds respectively compared to native EB (Figure 7A, P<0.01 and P<0.05 respectively). These results were consistent with the qRT-PCR observation that the dSPT seeded ECM constructs express higher Brachyury than dRA seeded constructs. Collectively, these expression profiles suggest the decellularized EB scaffolds enhanced ESC differentiation as demonstrated by upregulation of gastrulation-related genes and downregulation of pluripotency marker Nanog.

## Discussion

Dynamic reciprocity between cell and ECM is known to trigger cellular differentiation and determine cell fate commitment. Integrins on cells also appear to change to dictate the matrix preference as cells differentiate towards specific lineage [34]. The conventional cell culture and tissue engineering scaffolds, however, use single purified proteins and do not mimic the complexity of the differentiating stem cell extracellular microenvironment. Recently there has been a shift towards multiple-matrix systems of purified proteins, which have been shown to improve stem cell proliferation and differentiation [35], [36]. Natural ECM derived from native tissues and organs can meet this requirement by providing a more physiologically relevant scaffold that recapitulates the complex *in vivo* microenvironment [18], [27], [37], [38], [39]. Here, we report that ECM components isolated from differentiating EBs can act as a natural ECM mimicking scaffold to support ESC functions, such as proliferation and differentiation.

In this study, we first evaluated the ECM composition of two different types of differentiating EBs (spontaneously differentiated and RA treated). RA is one of the most important morphogens in vertebrate ontogeny and can be used to induce neural differentiation of ESC *in vitro* [40], [41]. We wanted to examine the ECM components of both types of differentiating EBs to see how the ECM microenvironment of RA treated EBs differs compared to SPT EBs. Consistent with other findings, RA treatment promoted EB into neuroectodermal lineage accompanied by a different ECM milieu in comparison with SPT EBs. SPT EBs formed more cystic cavities than the RA treated EBs (Figure 1D, E). The cavitation process occurs in the earliest developmental stage just prior to the start of gastrulation [42]. The coordination event that leads to cavitation is a basement membrane (BM)-dependent mechanism [42]. The BM coordinates both epithelialization and apoptosis throughout development whenever a lumen or cavity is to be created [43], [44]. Therefore this likely led to higher expression of BM proteins such as Collagen I, IV and Laminin found in the SPT EBs (Figure 2A) where more cavitation is found. Moreover, one recent report also shows that EBs containing RA treated microspheres resulted in down-regulation of matrix molecules such as collagen fibronectin, vitronectin, osteopontin and versican [45] which is consistent with our findings to show that our RA treated EBs have lower overall ECM expression than SPT EBs (Figure 2C). Spatially, the distribution of the Fibronectin (FN) molecules was found more in the core region of the EB in SPT EBs but more on the peripheral regions of RA treated EBs. FN is involved in adhesion and migration of mesodermal, neural crest and primordial germ cells in vertebrate development [46], [47]. The localization of FN in the core region may be attributed to the guidance cues needed for cell migration for cavity formation in the SPT EBs. In contrast, in the RA EBs where less cavitation is observed - FN is less prominent around the core region.

Next, we isolated these unique complex assemblies of ECM molecules from native SPT EB and RA EB via detergent decellularization technique. Studies have shown that detergents perform differently depending on the types of tissue during decellularization process [48], therefore protocols require re-evaluation for each application. Here, we examined three different detergents - 1% Triton X-100, 0.1% Sodium Deoxycholate (DOC), and 0.1% Sodium dodecyl sulfate (SDS) - to select the best protocol for decellularization of EB. SDS and DOC are aggressive ionic detergents; they are very effective for removing cellular material, but they have been shown to denature ECM proteins [18]. However, some other reports suggest SDS and DOC decellularization to be milder than non-ionic detergent Trion X-100 [49], [50]. The key parameters for a successful decellularization protocol are 1) complete or near complete removal of native cellular materials [51], and 2) maintenance of key ECM proteins [48]. Our result shows that decellularization of EB with 0.1% of DOC and 1% Triton X-100 still retain residual nuclei staining from H&E sections. In contrast, 0.1% SDS gave the best result in nuclear content removal (Figure 3B). The remaining DNA content was also consistent with the minimal industrial standard of acellularity [26], [51] (>99% DNA material removal, Table 1). Given that 0.1% SDS satisfies first key criteria of decellularization, all of the subsequent experiments were performed using this detergent only. This requirement is instrumental because residual DNA fragments in decellularized ECM have been shown to elicit immunological response when used in clinical transplantation [51].

The second key parameter for successful decellularization is the preservation of ECM components. Our results indicated that both groups of decellularized EBs preserved the key ECM components (Figure 4A, B) and sulfated GAGs that are found in the native EBs (Table 3). One caveat from the processing of decellularized EB is the loss of spatial information. Spatial information in the native EBs was analyzed as single EBs, whereas in the case of decellularized EB scaffolds –multiple decellularized EBs were pooled together, centrifuged and analyzed. As a result, spatial information as seen in the native EB was lost in the decellularized EB. Furthermore, it results in compaction of the ECM and loss of porosity, which can be attributed to the lack of cellular infiltration during recellularization. Hence upon reseeding with ESCs, they were found growing on the surface, and minimally infiltrated to populate the interiors of the decellularized EB scaffold (Figure 6C).

A crucial step determining the success of novel materials in tissue engineering applications is the efficiency of cell seeding strategy. The goal is to achieve high efficiency of attachment to maximize the utilization of donor cells and scaffold, by designing a controlled reproducible cell seeding methodology. In this study, we report a novel cell seeding strategy, inspired by the technique of hanging drop cultures. This technique allows uniform seeding of cells onto scaffolds by dispensing equal numbers of ESC in physically separated droplets of media onto decellularized EB scaffolds suspended from the lid of a Petri-dish. The cells are forced to “see” the scaffold based on the concept of forced proximity of cells to scaffold which in turn encourages the cells to attach to the scaffold. Our decellularized EB scaffold being small enough (∼2 mm) to fit within small volume of medium (usually less than 50 µL), this technique allows maximum seeding efficiency utilizing only a defined and low number of cells.

Differences between ECM components of dSPT and dRA was not detectable by IHC, but other quantitative assays such as Sircol collagen and Blyscan sGAG revealed that dSPT has a higher ECM content than dRA EB scaffold (Table 3, 4). This suggests that there could be micro-niches available at only cellular detection level and our IHC assays are not sensitive enough to pick up the differences. In addition, since dSPT EB scaffolds have a higher total protein content retention (Table 2) than dRA EB scaffold; this could result in more undefined cellular binding motifs for cellular attachment, which in turn could lead to a cascade of downstream cell signaling activation including proliferation and differentiation.

To test this hypothesis, we examined the ESC functions in terms of proliferation and differentiation after seeding and culture on decellularized EB scaffolds for 6 days. Our result shows that ESC seeded on dSPT EB scaffold behaved differently than ESC seeded on dRA scaffold. Higher cellular attachment and proliferation were observed when ESCs are seeded on dSPT EB scaffold compared to dRA EB scaffold. Differentiation functions as evaluated by qRT-PCR also show the direct impact of ECM from decellularized EB on enhancing differentiation of ESC. As ESC differentiate, they activate a well-conserved cascade of genes that govern the earliest events of gastrulation and germ layer formation [52]. An early marker of this process is expression of the prototypical primitive streak gene Brachyury. Our gene expression analysis and protein expression both confirmed higher upregulation (5.9-fold) of Brachyury level when ESC are seeded on dSPT ECM scaffold, modest upregulation (3.9-fold) on dRA EB scaffold compared to native EB. T box gene Brachyury is expressed in the trophectoderm-derived extraembryonic ectoderm of the mouse embryo [53], [54], and it is later involved in embryonic hemopoiesis and vasculogenesis. Transcription of Brachyury in ESC seeded EB scaffolds, which contain no trophectoderm derivatives, may be connected to the ESC's propensity for hemopoiesis and vasculogenesis. Other early gastrulation event related genes examined (FGF5 and FGF8) were also upregulated in cell-dSPT construct with the exception for AFP which is associated with VE differentiation. These results are consistent with other reports that show exogenous ECM such as Matrigel blocked formation of VE but enhanced mesoderm formation in mouse EBs [55] and human EBs [56]. Overall, differentiation of the ESCs on dSPT resulted in much stronger germ layer expression than dRA cell seeded scaffold.

During ESC differentiation, the ECM dynamically changes and remodels to accommodate for the alterations of cellular phenotype and differentiation. In theory, this will give rise to the desired differentiation niche that best fits, promotes and matures the ESCs to organized and functional tissues. Hence, It is intuitive to think that the neuroectodermal committed EB would give rise to a decellularized ECM microenvironment that favors neuroendodermal lineage commitment. However, our result did not support that claim. Neuroectoderm expression - Nestin was much lower in dRA seeded construct compared to dSPT seeded construct and native EB (control) (Figure 7A) One possible explanation is that neural progenitor cells do not produce appreciable ECM [57], [58], [59]. Increased number of Nestin positive cells in the RA treated EB suggests higher numbers of neural progenitor cells at the cost of other ECM producing resident populations (Figure 1L). As a result we observed an overall lower secretion of ECM content (Col I, Col IV and Laminin) in the RA treated EB (Figure 2C), which in turn gave rise to decellularized EB scaffold with less ECM proteins. Laminins are the major non-collagenous glycoproteins of all basal laminae and it has been implicated important in neurogenesis [60]. Mouse and human neural stem cell precursors differentiate into neuronal lineage on laminin but not fibronectin [61], and they also responds to laminin in a dose-dependent manner [60]. Lower laminin levels observed in the native RA treated EB (Figure 2C) could have led to decreased neuroectoderm differentiation when ESCs were seeded on dRA ECM scaffolds. It is noteworthy that certain cells types are unable to secret ECM; instead, they rely on neighboring cell populations to provide their supporting ECM niche. Pancreatic islets and neuronal cells are some examples of these cell types [58].

In contrast, the ECM from SPT EB with high Brachyury level in the native form was able to induce the same lineage of differentiation (mesoderm) when seeded back with ESCs (Figure 7A). During gastrulation, ECM proteins are mainly deposited by the primitive mesendoderm [62] – this probably permits the establishment of higher ECM content in the SPT EBs where onset of higher Brachyury level is observed (Figure 1H). Hence, decellularized version of SPT EB will likely retain the favorable ECM microenvironment that is conducive of mesodermal lineage derivatives.

Functional differences in differentiation assays were observed between ESC seeded on dSPT and dRA scaffolds despite only marginal differences detected in the ECM compositions with the assays performed. It is possible to perform more sensitive assays (eg. proteomic mass spec comparisons) to provide more thorough characterization of the ECM compositions. Importantly, it is likely that having only the appropriate ECM milieu may not be sufficient to dictate cell fate specification but mandate the synergistic coupling of soluble growth factors to drive lineage commitment. This is especially applicable for cell types that do not produce their own ECM (eg. Neural progenitor cells), and become more dependent on growth factors for lineage specification. In contrast, cell fate commitment on cell types that do produce their own ECM (eg. mesendoderm lineages), the resulting ECM microenvironment is likely to be conducive to give rise to the same lineage specification as demonstrated by our recellularized dSPT EB scaffolds (Figure 7A). In our study, both ESC-seeded EB scaffolds were cultured in differentiation medium without LIF to allow spontaneous differentiation. No chemical inductions were introduced to drive the differentiation in order to investigate the effects solely exerted by ECM. In future studies, coupling of growth factors with the EB ECM scaffolds could help establish better differentiation efficiency, and yield insights into the key combination regulators of the system.

## Conclusions

Decellularization of EB with 0.1% SDS efficiently removes cells while preserving major ECM proteins. This bioactive EB scaffold is supportive of ESC proliferation, and enhanced early differentiation when seeded with ESC. Decellularized EB scaffolds represent a candidate of three-dimensional bioactive scaffold that mimics natural ECM from an embryonic source that can potentially be utilized as a biomaterial to mediate ESC functions.

## Supporting Information

Figure S1 **Effects of different concentrations of RA on EB.** (A) Morphological analysis demonstrated different sizes of EBs, and also (B) different Nestin expression level resulted from different concentrations of RA treatment.
(TIF)

Figure S2 **Gene expression of the seeded EB constructs after 6 days of culture.** (A) qRT-PCR result at day 6 to depict gene expressions related to early gastrulation. The expression level is normalized to undifferentiated ESC.
(TIF)
