# Correction: Extracellular Matrix Aggregates from Differentiating Embryoid Bodies as a Scaffold to Support ESC Proliferation and Differentiation

**DOI:** 10.1371/annotation/201f73d1-bf44-4528-b71c-b537aad520f3

**Published:** 2013-10-28

**Authors:** Saik-Kia Goh, Phillip Olsen, Ipsita Banerjee

A funding organization was incorrectly omitted from the Funding Statement. The following sentence should be included with the Funding Statement: "Co-author Phillip Olsen was supported partially by the Research Experiences for Undergraduates (REU) Program of the National Science Foundation."

In addition, the following sentence should be read with the Acknowledgments: "Authors would like to acknowledge Mutant Mouse Regional Resource Center U42OD010918 for providing mouse strain 011981-MU [Nagy ES cell line R1 with EGFP (B5)]."

