# Supplementary figures and images for: Extracellular Matrix Aggregates from Differentiating Embryoid Bodies as a Scaffold to Support ESC Proliferation and Differentiation
Source: PLoS One. 2013 Apr 18;8(4):e61856. doi: 10.1371/journal.pone.0061856 (PMC3630218; doi:10.1371/journal.pone.0061856)

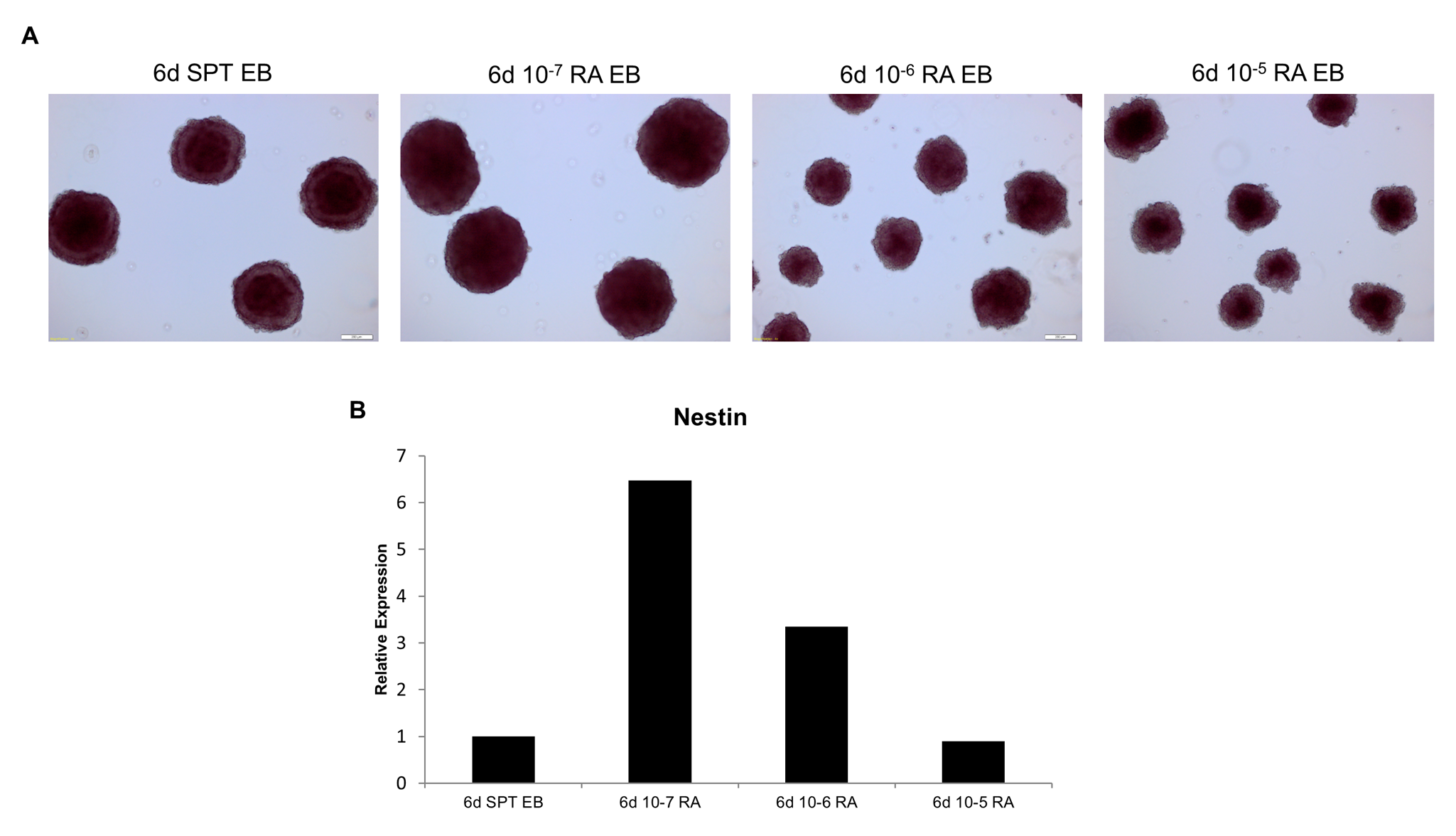

Supplement: Figure S1 — Effects of different concentrations of RA on EB. (A) Morphological analysis demonstrated different sizes of EBs, and also (B) different Nestin expression level resulted from different concentrations of RA treatment. (TIF) [file pone.0061856.s001.tif]

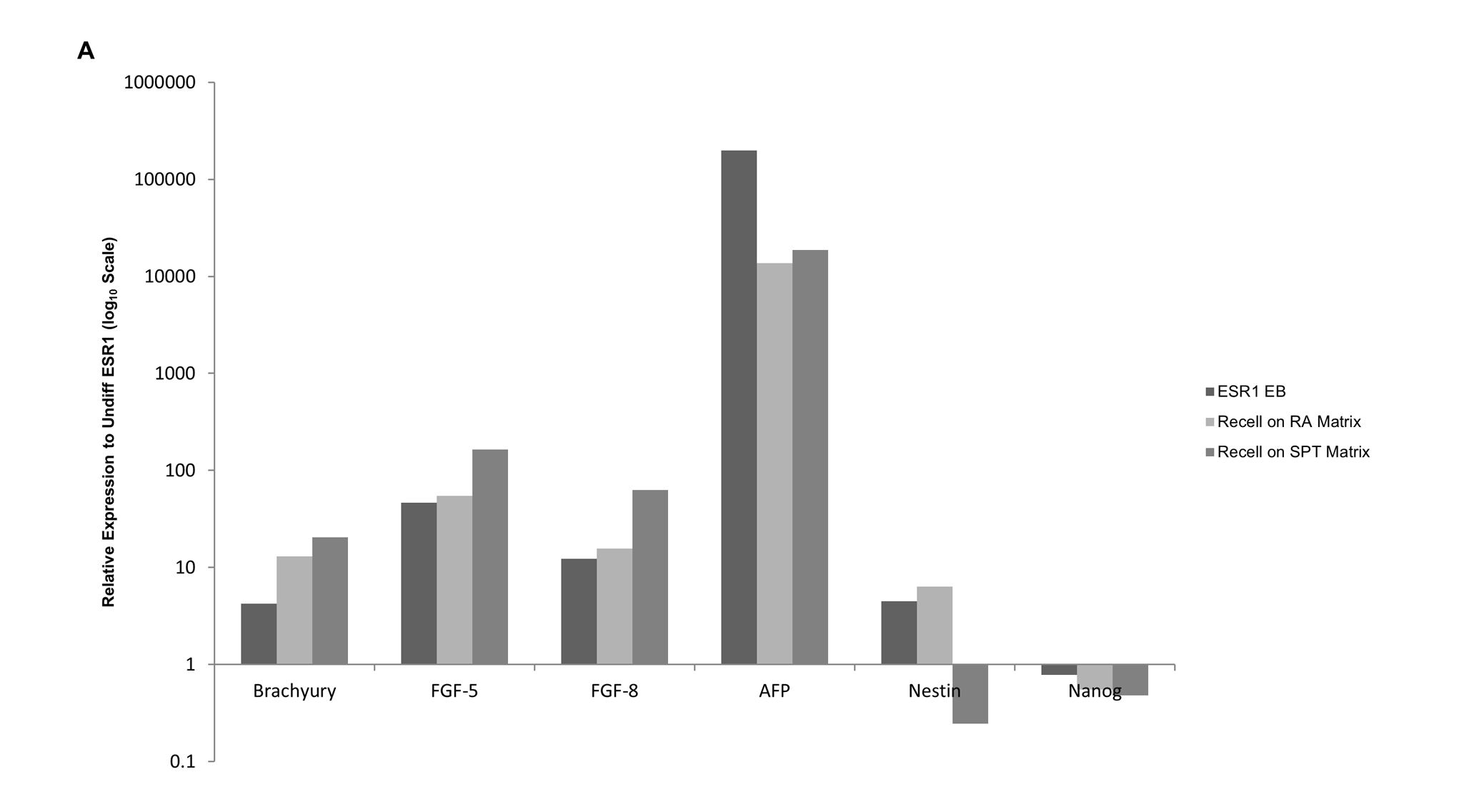

Supplement: Figure S2 — Gene expression of the seeded EB constructs after 6 days of culture. (A) qRT-PCR result at day 6 to depict gene expressions related to early gastrulation. The expression level is normalized to undifferentiated ESC. (TIF) [file pone.0061856.s002.tif]
